# Phosphotungstic acid heterogenized by assembly with pyridines for efficient catalytic conversion of fructose to methyl levulinate†

**DOI:** 10.1039/c8ra02278j

**Published:** 2018-05-04

**Authors:** Chengjiang Fang, Yan Li, Wenfeng Zhao, Weibo Wu, Hu Li, Chao He, Song Yang

**Affiliations:** State Key Laboratory Breeding Base of Green Pesticide & Agricultural Bioengineering, Key Laboratory of Green Pesticide & Agricultural Bioengineering, Ministry of Education, State-Local Joint Laboratory for Comprehensive Utilization of Biomass, Center for Research & Development of Fine Chemicals, Guizhou University Guiyang Guizhou 550025 China jhzx.msm@gmail.com hli13@gzu.edu.cn +86(851)8829-2170 +86(851)8829-2171; School of Environmental Science and Engineering, Sun Yat-sen University Guangzhou 510006 P. R. China

## Abstract

Solid acid-catalyzed sugar degradation has been considered to be an efficient approach to synthesize alkyl levulinates (which can be used as fuel additives and surfactants). However, those catalytic processes typically involve harsh reaction conditions and high cost for catalyst preparation. We prepared a series of phosphotungstic acid organic hybrids by a simple solvothermal method, and used them as heterogeneous catalysts for the selective degradation of fructose to methyl levulinate (ML) in methanol with high efficiency under mild reaction conditions. The catalysts were characterized systematically, and the effects of different substituents in pyridine, reaction temperature/time, catalyst dose, and fructose concentration studied. The 3-FPYPW hybrid prepared from 3-fluoropyridine and phosphotungstic acid exhibited superior catalytic activity for the synthesis of ML (82.5%) from fructose (97.8%). A possible reaction pathway was proposed. In addition, the catalyst could be separated from the reaction mixture readily, and reused without remarkable loss of reactivity.

## Abstract 2

A high yield of methyl levulinate (82.5%) was achieved from fructose *via* a one-pot multi-step conversion process using acidic 3-FPYPW as a heterogeneous catalyst.

## Graphical abstract

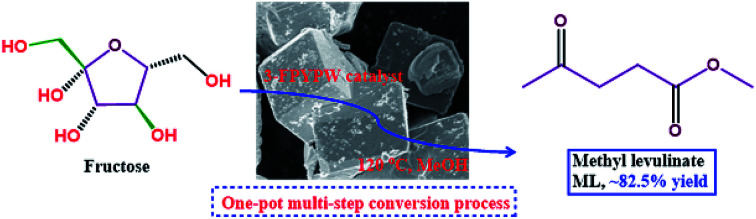

## 1. Introduction

With the depletion of fossil resources and progressive environmental damage, abundant and renewable resources have been explored to replace non-renewable petroleum sources for the sustainable supply of fuel and chemicals.^1–5^ In contrast to other renewable energies such as solar, wind and hydro power, biomass is the only feedstock that provides sustainable organic carbon resources for both biofuels and chemicals.^6–10^ In this respect, fructose, as an ample and inexpensive six-carbon sugar molecule, has been employed widely for the synthesis of bio-based products. For example, the loss of three water molecules from fructose catalyzed by an acid can yield 5-hydroxymethylfurfural, followed by alcoholysis to give alkyl levulinates. Among these levulinates, methyl levulinate (ML) has been identified as an important bio-platform compound with several excellent features, such as non-toxicity, high lubricity, flashpoint stability and good flow properties.^11,12^ ML has been used as an additive for gasoline and diesel, as well as for the flavoring and fragrances or as substrate for different types of condensation and addition reactions.^13–16^ Therefore, the development of efficient catalytic systems for direct conversion of fructose to ML is important.

Various acidic catalysts (*e.g.*, SO_4_^2−^/TiO_2_, H-USY(6), HPW, sulfated TiO_2_–ZrO_2_ and 5-Cl-SHPAO) have been reported to be efficient for the synthesis of ML from fructose.^17–21^ In particular, phosphotungstic acid (HPW) exhibits pronounced catalytic activity for the conversion of fructose to ML. Nevertheless, HPW has drawbacks, such as complete solubility in polar solvents, difficulty in separation, equipment corrosion, and environmental pollution. In general, the immobilization of HPW to prepare heterogeneous counterparts has been demonstrated to be an effective approach to overcome these drawbacks. For instance, a ML yield of 73.7% has been obtained from fructose in methanol using Fe-HPW-1 as a catalyst at 130 °C and 2 MPa for 2 h, and the ML yield dropped to 60% after repeat use of the catalyst five times.^22^ Lai and co-workers reported on the synthesis of ML from fructose in methanol at 180 °C for 1 h in the presence of 4-HPWFe-MMTSi catalyst: the ML yield reached 74%, and this catalyst exhibited good reusability.^23^ Although they have excellent catalytic performance, these catalysts have their own drawbacks, such as high temperature and pressure, the high cost of preparation, and several synthetic steps.

As a promising alternative, the assembly of HPW with basic organic species is an effective way to solidify the homogeneous acid and “tune” its acidity. For example, a series of acidic ionic liquid catalysts (*e.g.*, [MIMBS]_3_PW_12_O_40_, [MIMPS]_3_PW_12_O_40_, and [3·2H]_3_[PW_12_O_40_]_2_) were developed for the heterogeneous catalytic conversion of fructose into alkyl levulinates, 5-(ethoxymethyl)furfural and 5-hydroxymethylfurfural (HMF), in high yields (70–99%).^24–26^ In our previous study, an acidic hybrid catalyst (PYPW-1) was obtained by self-assembly of pyridine (PY) with HPW at room temperature in ethanol solution. It exhibited good reactivity in the catalytic conversion of HMF to 5-ethoxymethylfurfural (EMF) with 90% yield, but was nonselective for catalytic degradation of fructose (up to *ca*. 40% selectivity toward EMF).^27^

**Scheme 1:**
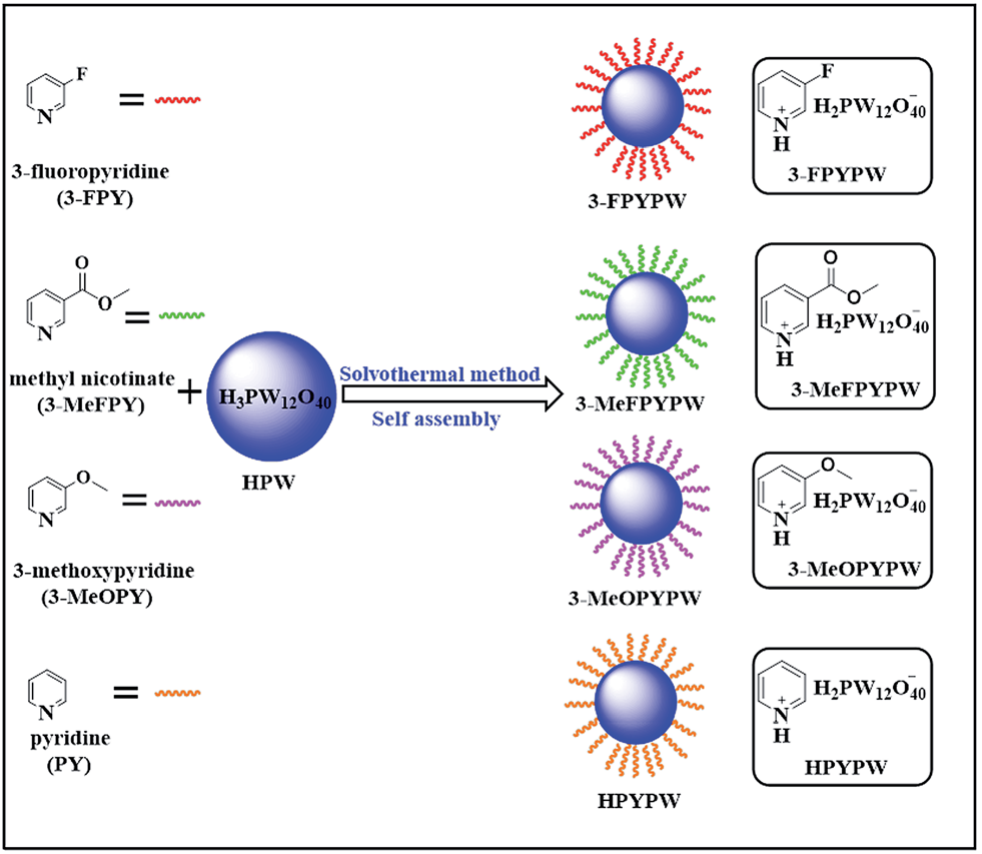
Preparation of various acidic hybrids (PYPWs) by assembly of HPW with pyridine under solvothermal conditions (schematic).

In the present work, a series of novel substituted pyridine phosphotungstates (PYPWs) were prepared using a renewed but simple solvothermal method (Scheme 1), wherein the type of substituted groups was correlated directly with the acid properties of the catalysts. The catalytic performance of these catalysts was evaluated, and found to be efficient for the direct conversion of fructose to ML in a one-pot process under relatively mild reaction conditions. The effects of different types of catalysts, catalyst dose, and reaction temperature/time on the conversion fructose to ML, as well as catalyst reusability, were investigated. Moreover, the possible pathway on the transformation of fructose into ML was proposed.

## 2. Experimental section

### 2.1 Materials

3-Fluoropyridine (3-FPY) (98.0%), methyl nicotinate (3-MeFPY) (99.0%), 3-methoxypyridine (3-MeOPY) (98.0%), pyridine (PY) (98.0%), fructose (99.0%) and phosphotungstic acid (HPW) (99.0%) were purchased from Beijing InnoChem Science & Technology. Other reagents used in this work were purchased from Beijing Maya Reagents; they were of analytical grade and not purified, unless stated otherwise.

### 2.2 Catalyst preparation

A 3-FPYPW sample was prepared using a solvothermal method. In a typical procedure, HPW (1 mmol) was added to absolute ethanol (32 mL) in a 100 mL stainless-steel autoclave containing a Teflon™-lined reactor, and stirred until complete dissolution at room temperature to obtain HPW solution, followed by addition of 3-FPY (1 mmol) with continuous stirring for 1.5 h at room temperature. The resultant precipitate was aged for 12 h at 90 °C, followed by filtration, washing with absolute ethanol, and drying at 80 °C overnight to give the solid acidic catalyst 3-FPYPW. For comparison, 3-MeFPYPW, 3-MeOPYPW, and HPYPW were also synthesized from HPW and the corresponding pyridine with different substituent groups (3-MeFPY, 3-MeOPY and PY) using the same method, respectively.

### 2.3 Catalyst characterization

The X-ray diffraction (XRD) patterns of the catalysts were recorded on an X-ray diffractometer (D8 Advance) with Cu Kα radiation (*λ* = 0.1548 nm) and a 2*θ* ranging from 5° to 80°. Fourier transform infrared spectroscopy (FT-IR) was recorded on a FT-IR spectrometer (NICOLET iS50) in KBr disks containing a specific amount of solid sample (*ca*. 10 mg). Scanning electron microscopy (SEM) was carried out using an electron microscope (FESEM XL-30; Philips). Transmission electron microscopy (TEM) images were obtained on a G2 F20 (FEI TECNAI) system. Thermogravimetric analysis (TGA) was conducted using an STA409 instrument in dry air at a heating rate of 10 °C min^−1^. Scanning transmission electron microscopy-high-angle annular dark-field (STEM-HAADF) imaging was done using an aberration-corrected G2 F30 S-TWIN system (FEI TECNAI) operating at 300 kV. The W elemental composition of 3-FPYPW catalyst was analyzed by inductively coupled plasma-optical emission spectrometry (ICP-OES) on an Optima 5300 DV instrument (PerkinElmer). The pore size of the 3-FPYPW sample was measured at 77 K on an ASAP 2460 analyzer (Micromeritics).

### 2.4 Analyses of the acid density of catalysts

The total acid density of solid catalysts was determined by the sodium ion-exchange method.^28^ Briefly, 0.05 g of solid acid catalyst was added to 2 mol L^−1^ NaCl solution (15 mL) followed by ultrasonic oscillation for 0.5 h. The resulting mixture was filtered and washed thrice with 2 mol L^−1^ NaCl solution. Afterwards, phenolphthalein and 0.1 mol L^−1^ NaOH solutions were employed as indicator and titrant, respectively. When the solution changed from colorless to light-red for 30 s, the endpoint of titration was reached. An expression for the accurate quantity of acid could be written as:

1 *c*(H^+^) = (*c*(OH^−^) × Δ*V*)/*m*

where *c*(H^+^) denotes the acid quantity of the catalyst (mol g^−1^), *c*(OH^−^) denotes the concentration of the NaOH solution (mol L^−1^), Δ*V* denotes the volume of the NaOH solution consumed in titration (L), and *m* represents the quality of the catalyst samples (g). The content of H^+^ was obtained using the following formula:

2 *n*(H^+^) = *c*(H^+^) × *m*

where *n*(H^+^) denotes the molar content of H^+^ (mmol), *c*(H^+^) denotes the acid quantity of the catalyst (mmol g^−1^) and *m* represents the amount of the catalyst in the reaction system (g).

### 2.5 Catalytic conversion of fructose into ML

The synthesis of ML from fructose was conducted in a 15 mL Ace pressure tube equipped with a magnetic stirring bar and placed in an oil bath. In a general synthetic process, 1 mmol fructose, 90 mg 3-FPYPW catalyst (containing 0.1 mmol H^+^), and 5 mL methanol were added to the tube while the reaction temperature and time were regulated within the desired range. After the reaction, the tube was cooled to room temperature with flowing water. The reaction solution was filtered to remove solid catalyst, and transferred into a volumetric flask and diluted with methanol for subsequent quantitative analyses.

### 2.6 Catalyst recycling

Recycling of 3-FPYPW catalyst was examined in methanol (5 mL), with 3-FPYPW (0.1 mmol H^+^) and fructose (1 mmol) for 10 h at 120 °C. After each cycle of the reaction, the catalyst was separated by centrifugation from the reaction mixture, followed by washing thrice with absolute ethanol, and drying for 24 h at 80 °C. The recovered catalyst was used directly for the next cycle under identical reaction conditions.

### 2.7 Analytical method

The HMF concentration was analyzed by high-performance liquid chromatography (HPLC) using an 1100 (Agilent Technologies) system fitted with a Lichrospher C18 column and UV detector (*λ* = 284 nm). The column temperature was set at 25 °C, and the mobile phase used was acetonitrile/0.1 wt% acetic acid aqueous solution (30 : 70, *v/v*) at a flow rate of 1.0 mL min^−1^. The concentration of fructose was determined by HPLC using an Aminex HPX-87H column (Bio-Rad Laboratories) and a refractive index (RI) detector. The column temperature was set at 65 °C, whereas the mobile phase was CH_3_CN/H_2_O (80/20, *v/v*) at a flow rate of 1.0 mL min^−1^. ML was analyzed by gas chromatography using an 7890B (Agilent Technologies) system fitted with a HP-5 column (30 m × 0.320 mm × 0.25 μm) and a flame ionization detector (FID); naphthalene (10 mg) was added into liquid products as an internal standard. The amount of byproducts (MMF, DMMF) was measured by gas chromatography-mass spectrometry (GC-MS) using a 6890N GC/5973 MS system (Agilent Technologies), and quantified by area normalization. The conversion rate (*X*, mol%) of fructose, and HMF or ML yield (*Y*, mol%) were calculated using the following equations:

3 *X* (%) = [1 − (molar concentration of fructose in products)/(molar concentration of initial fructose)] × 100%

4 *Y* (%) = (molar concentration of product)/(molar concentration of initial fructose) × 100%

## 3. Results and discussion

### 3.1 Catalyst characterization

**Fig. 1:**
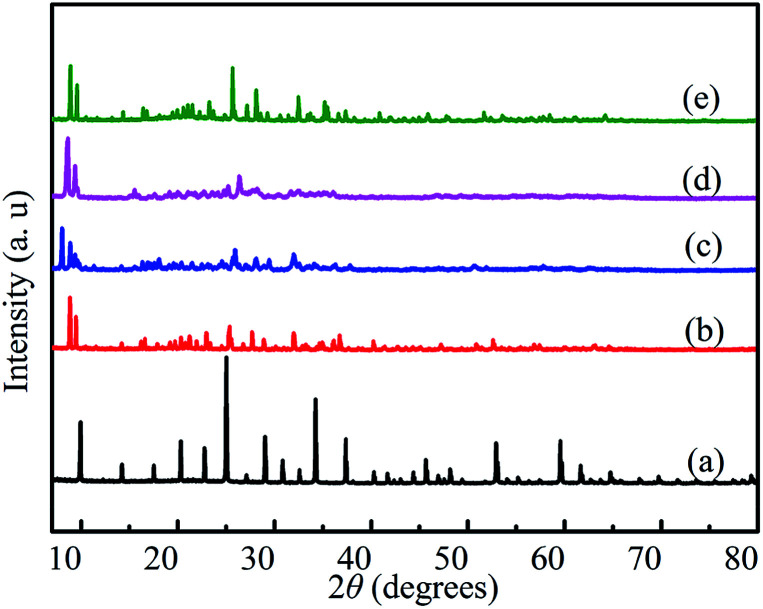
XRD patterns of (a) HPW, (b) HPYPW, (c) 3-MeOPYPW, (d) 3-MeFPYPW and (e) 3-FPYPW.

The XRD patterns of HPW, HPYPW, 3-MeOPYPW, 3-MeFPYPW and 3-FPYPW are provided in Fig. 1. HPW displayed the typical cubic secondary structure of a Keggin anion with characteristic diffraction peaks positioned at 9.9°, 14.1°, 17.6°, 20.3°, 22.7°, 24.8°, 29.1° and 34.1°.^29^ The XRD patterns of 3-FPYPW, 3-MeFPYPW, 3-MeOPYPW, and HPYPW exhibited a similar structure to that of HPW: cubic. The diffraction peaks of 3-FPYPW, 3-MeFPYPW, 3-MeOPYPW and HPYPW appeared at relatively low peak intensity when the H^+^ of H_3_PW_12_O_40_ was substituted by 3-FPY, 3-MeFPY, 3-MeOPY and PY, respectively. For 3-FPYPW, 3-MeFPYPW, 3-MeOPYPW and HPYPW catalysts, the intensity of the diffraction peaks decreased gradually with the change of substituents, and the order of intensity was 3-FPYPW > 3-MeFPYPW > 3-MeOPYPW > HPYPW. The 2*θ* values shifted towards a lower angle upon introduction of organic ligands (3-FPY, 3-MeFPY, 3-MeOPY and PY) into HPW, thereby implying an electrostatic interaction between HPW and organic species.

**Fig. 2:**
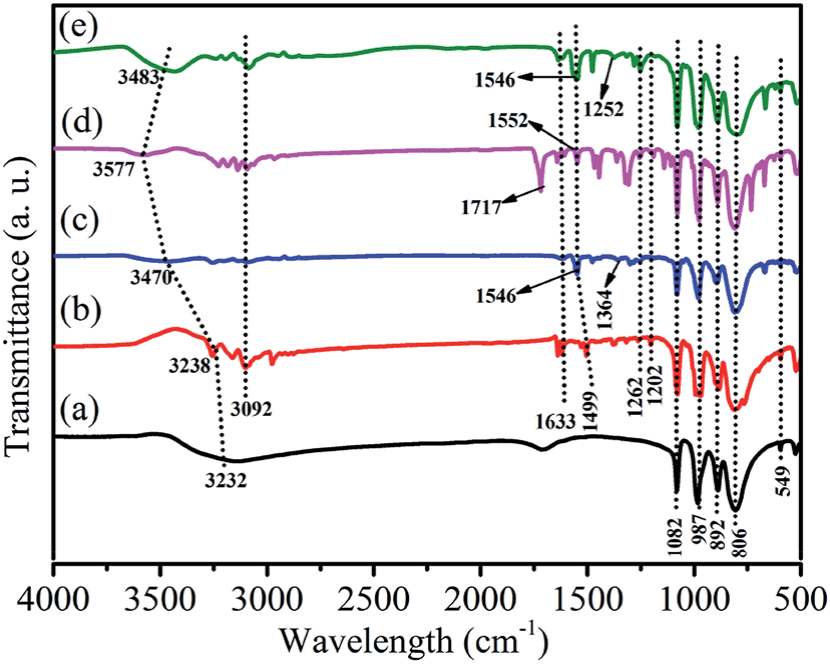
FT-IR spectra of (a) HPW, (b) HPYPW, (c) 3-MeOPYPW, (d) 3-MeFPYPW and (e) 3-FPYPW.

Fig. 2 shows the FT-IR spectra of HPW, HPYPW, 3-MeOPYPW, 3-MeFPYPW and 3-FPYPW. HPW and the hybrids exhibited four typical absorption peaks of a Keggin structure at 800–1100 cm^−1^, which could be assigned to the asymmetric vibrations of the W–O_c_ (806 cm^−1^), W–O_b_ (892 cm^−1^), W–O_d_ (987 cm^−1^) and P–O_a_ (1082 cm^−1^) bonds, respectively. Moreover, the peak at 594 cm^−1^ was due to the bending vibration of the P–O band. Absorption peaks at around 1633, 1499, 1262, 1202 and 3092 cm^−1^ were attributed to the *v*(C=C), *v*(C=N), *δ*(C–H), *γ*(C–H) and C–H bands of pyridine molecules, respectively. Correspondingly, the peak at 1364 cm^−1^, 1717 cm^−1^, 1252 cm^−1^ and 3232 cm^−1^ was assigned to the C–O band (Fig. 2(c)), C=O band (Fig. 2(d)), C–F band (Fig. 2(e)), and surface-adsorbed water, respectively. Upon introduction of the substituents (–OCH_3_, –COOCH_3_, –F) into pyridine, the characteristic peaks of pyridine shifted to a relatively higher wavenumber, which may have been due to the conjugation effect or induction effect.

**Fig. 3:**
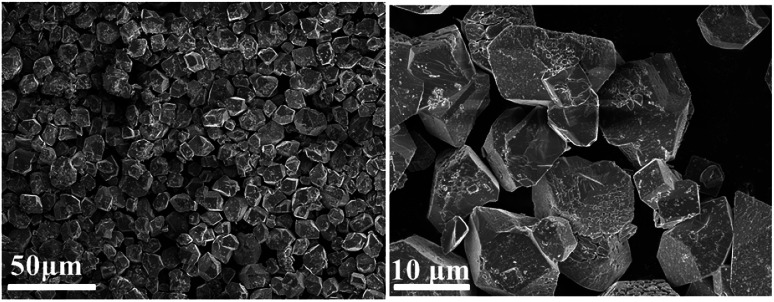
SEM of images 3-FPYPW catalyst.

**Fig. 4:**
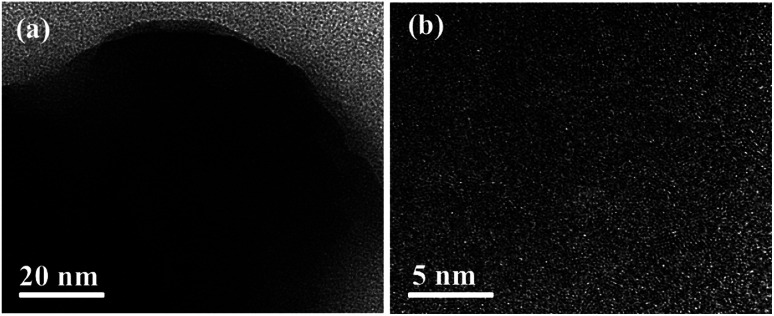
TEM images of 3-FPYPW catalyst.

**Fig. 5:**
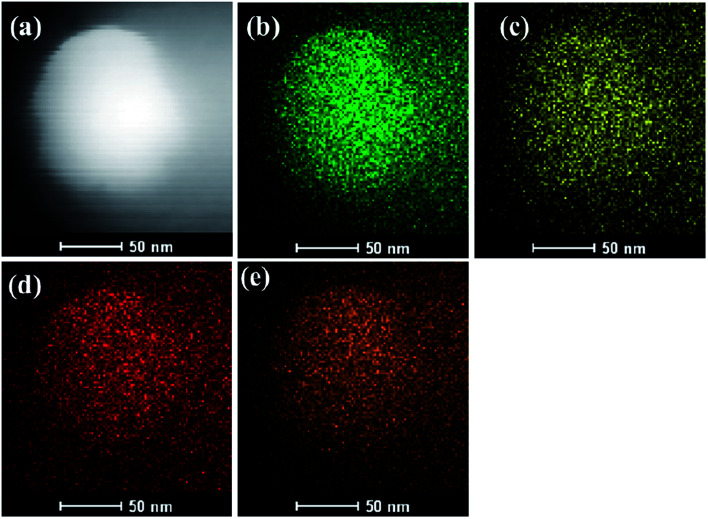
(a) STEM-HAADF image and (b) W, (c) P, (d) N and (e) F elemental mappings for 3-FPYPW catalyst.

The morphology of the 3-FPYPW catalyst was investigated by SEM. Fig. 3 shows that 3-FPYPW displayed a smooth polyhedron structure. The lattice plane spacing was observed in the TEM image of 3-FPYPW (Fig. 4(b)), and showed that 3-FPYPW catalyst was crystalline, data that matched closely with XRD results (Fig. 1(f)). In addition, the distribution of F, N, P and W elemental mappings of 3-FPYPW were analyzed by STEM-HAADF, as shown in Fig. 5(b–e). The results revealed a successful exchange of protons for 3-fluoropyridine, and these elements were well dispersed. Moreover, the acidity of catalysts was analyzed by the sodium ion-exchange method. With enhancement of the absorption capacity of the substituent group, the total acid density of the catalyst also increased. The acidity of the catalysts was in the order: 3-FPYPW (1.13 mmol g^−1^) > 3-MeFPYPW (0.95 mmol g^−1^) > 3-MeOPYPW (0.79 mmol g^−1^) > HPYPW (0.51 mmol g^−1^).

### 3.2 Catalyst screening for the conversion of fructose to ML

The degradation of fructose into ML in methanol was carried out using various catalysts (Table 1). The soluble catalyst HPW gave fructose conversion of 98.3% and ML yield of 83.5%. 3-FPYPW exhibited the best catalytic activity, and 82.5% yield of ML from fructose (97.8% conversion) was achieved for 10 h at 120 °C. In the presence of the 3-MeFPYPW sample, fructose conversion and ML yield were 87.8% and 56.4%, respectively. For the 3-MeOPYPW catalyst, 81.6% fructose conversion and 36.5% ML yield were obtained. For the conversion of fructose to ML, the range of catalytic activity was HPW (TON = 8.9) > 3-FPYPW (8.1) > 3-MeFPYPW (6.6) > 3-MeOPYPW (5.1) > HPYPW (3.6). These results implied that an appropriate increase in the acid density of the catalyst enhanced its catalytic efficiency for the synthesis of ML from fructose. The effect of the molecular structure of the various PY moieties containing different substituents in the catalysts on the conversion of fructose to ML was investigated. For HPYPW, 3-MeOPYPW and 3-MeFPYPW catalysts, the yield of ML increased with the increasing steric hindrance of substituents. The 3-FPYPW catalyst showed good catalytic activity in the conversion of fructose to ML because the –F substituent has strong electronegativity. A possible reason is that the structure of the catalyst changed due to steric hindrance or the induction effect of the substituents. To elucidate this phenomenon further, a series of catalysts were tested by N_2_ adsorption–desorption isotherms. The results showed that the pore sizes of 3-FPYPW, 3-MeFPYPW, 3-MeOPYPW and HPYPW catalysts were 9.0 nm, 8.4 nm, 6.6 nm and 5.5 nm, respectively, demonstrating that the molecular structure of the catalyst was closely related to the yield of ML.

**Table 1:** Conversion of fructose to ML using various catalysts^a^

Catalyst	ML (*Y*, %)	HMF (*Y*, %)	DMMF (*Y*, %)	MMF (*Y*, %)	Fructose (Conv. %)	TON^b^
HPW	83.5	0	0	0	98.3	8.9
3-FPYPW	82.5	0	0	0	97.8	8.1
3-MeFPYPW	56.4	0.5	8.3	11.6	87.8	6.6
3-MeOPYPW	36.5	2.3	10.8	22.7	81.6	5.1
HPYPW	16.4	2.0	11.2	11.5	55.7	3.6

a Reaction conditions: fructose (0.2 mol L^−1^), catalyst (90 mg), 5 mL methanol, 120 °C, 10 h.

b Turnover number (TON) was evaluated on the basis of the molar amount of ML/mole effective acid sites.

### 3.3 Effect of catalyst dose on the synthesis of ML from fructose

**Fig. 6:**
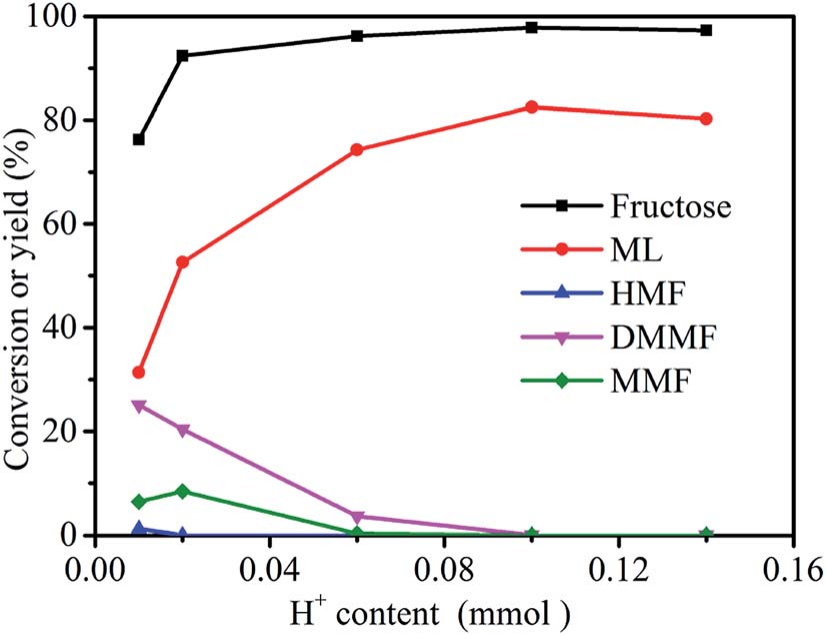
Effect of H^+^ content on the synthesis of ML from fructose. Reaction conditions: fructose (0.2 mol L^−1^), 5 mL methanol, 120 °C, and 10 h.

The effect of catalyst dose (evaluated by H^+^ content, see Section 2.4) on the conversion of fructose to ML was investigated using 3-FPYPW as catalyst (Fig. 6). The yield of ML increased from 31.4 to 82.5% by increasing the H^+^ content in 3-FPYPW from 0.01 to 0.1 mmol. Meanwhile, the yields of the intermediates HMF, MMF and DMMF were reduced gradually to 0 with an increase in catalyst dose from 0.01 to 0.1 mmol H^+^. Upon further increase of the H^+^ content to 0.14 mmol, the yield of ML decreased to 80.3% due to side reactions, as clarified by GC-MS (Fig. S3–S6†). Therefore, 3-FPYPW containing 0.1 mmol H^+^ was chosen as the optimum amount of catalyst.

### 3.4 Effect of reaction temperature/time on the conversion of fructose to ML

**Fig. 7:**
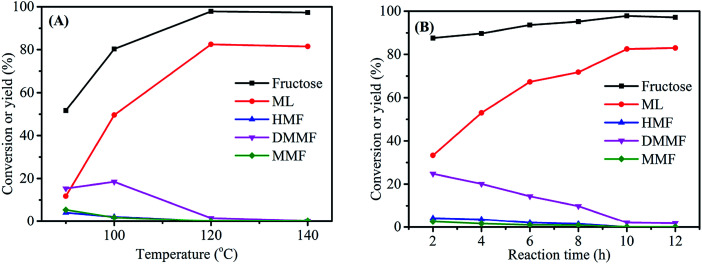
(A) Effect of reaction temperature on fructose conversion. Reaction conditions: 0.2 mol L^−1^ fructose, 5 mL methanol, 3-FPYPW (0.1 mmol H^+^), and 10 h. (B) Effect of reaction time on fructose conversion. Reaction conditions: 0.2 mol L^−1^ fructose, 5 mL methanol, 3-FPYPW (0.1 mmol H^+^), and 120 °C.

The reaction temperature was an important parameter in the synthesis of ML from fructose. Experiments were carried out at four reaction temperatures (90, 100, 120 and 140 °C) using fructose (0.2 mol L^−1^), 3-FPYPW (0.1 mmol H^+^) and 10 h to further optimize the yield of ML (Fig. 7(A)). When the temperature was increased from 90 to 120 °C, the ML yield rose correspondingly from 11.8 to 82.5%, with the yields of intermediate products decreasing to 0. However, when the reaction temperature continued increasing to 140 °C, almost no change in the conversion of fructose was observed, but the ML yield began to fall. This may have been due to side-reactions at high temperature. In addition, the effect of the reaction time on the yield of ML was investigated under optimized reaction conditions of fructose (0.2 mol L^−1^), 3-FPYPW (0.1 mmol H^+^), and 120 °C. As shown in Fig. 7(B), the yield of ML increased from 33.3 to 82.5% by prolonging the reaction time from 2 to 10 h. Then, the yield of ML was not changed significantly when the reaction time reached 12 h. In addition, at a reaction temperature of 140 °C, different reaction times (1 h, 2 h, 4 h, 6 h and 8 h) were selected for investigation (Fig. S1†). The results indicated that with extension of the reaction time from 1 to 8 h, the yield of ML increased from 43.8 to 80.2%. The maximum ML yield was comparable with that (82.5%) obtained for 10 h at 120 °C. Based on energy consumption, 120 °C and 10 h were, thus, the optimum reaction temperature and time for the conversion of fructose to ML, respectively.

### 3.5 Effect of fructose concentration on the conversion of fructose to ML

**Fig. 8:**
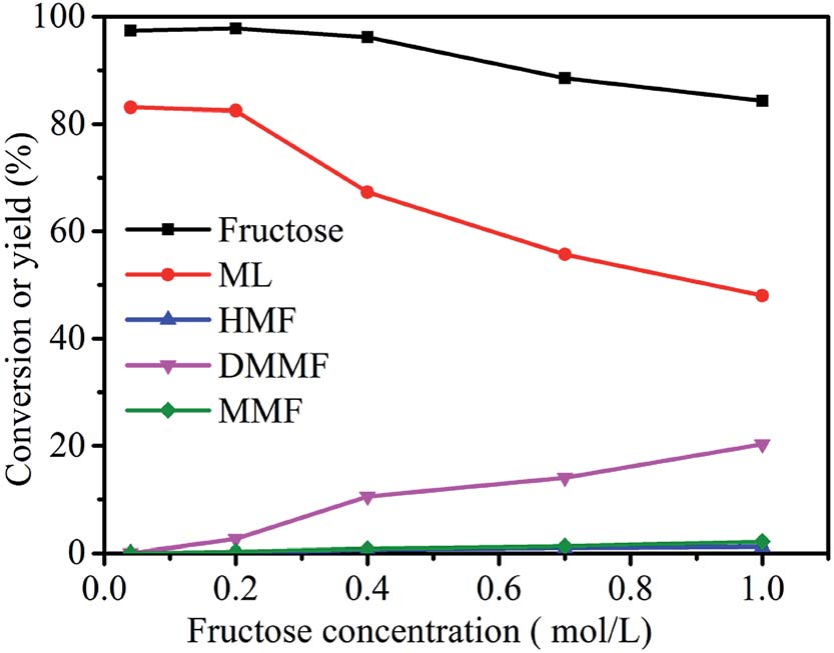
Effect of fructose concentration on the synthesis of ML from fructose. Reaction conditions: 5 mL methanol, 3-FPYPW (0.1 mmol H^+^), 120 °C, and 10 h.

As shown in Fig. 8, the fructose concentration (0.04, 0.2, 0.4, 0.7 and 1 mol L^−1^) had a very important role in the synthesis of ML from fructose. When the substrate concentration was 0.04 mol L^−1^, the conversion of fructose was 97.4%, and the ML yield reached 83.1%. With a decrease in fructose concentration, the yield of ML and conversion of fructose showed no significant increasing trends. The ML yield decreased from 82.5 to 48.0% when the fructose concentration increased from 0.2 to 1.0 mol L^−1^. These results suggest that relatively higher fructose concentrations led directly to a decrease in the yield of ML, which could have been due to a lack of active sites in the catalyst.^16,30^ Herein, the optimum fructose concentration was set at 0.2 mol L^−1^.

### 3.6 Catalyst recycling

**Fig. 9:**
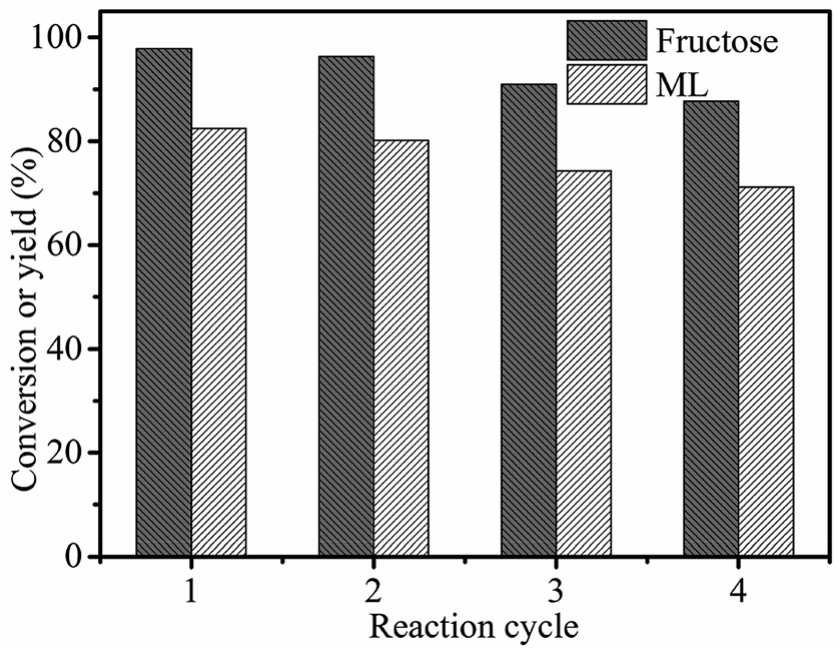
Recyclability of 3-FPYPW in the conversion of fructose to ML. Reaction conditions: 0.2 mol L^−1^ fructose, 5 mL methanol, 3-FPYPW (0.1 mmol H^+^), 120 °C, and 10 h.

**Fig. 10:**
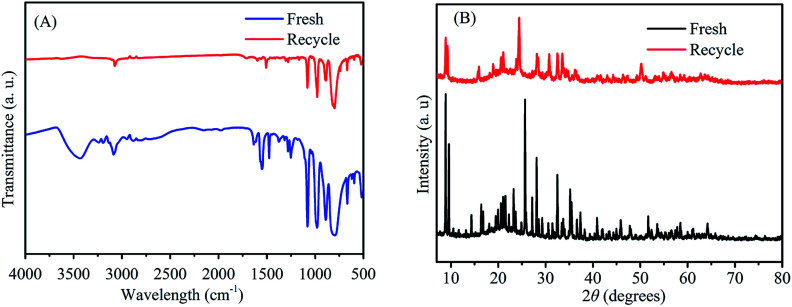
(A) FT-IR spectra and (B) XRD patterns of recycled (after the fourth run) and fresh 3-FPYPW catalysts.

To investigate the reusability of 3-FPYPW catalyst, a four-cycle experiment was carried out under optimum reaction conditions. The recovery of 3-FPYPW catalyst was 96.7%; hence the leaching rate was 3.3% relative to the initial dose of the catalyst after reusing four times. To investigate further the nature of the catalyst, the leaching experiment was undertaken according to specific steps. Briefly, the mixture was cooled to room temperature and filtered after reacting for 3 h. The filtrate was mixed further with 90 mg fructose and reacted at the same temperature (120 °C) for another 1 h. The yield of ML was increased by only 0.6%. This result showed that only a small amount of 3-FPYPW was leached into the mixture after the reaction. The filtrate mentioned above was tested further by ICP-OES, and only 0.4% of W in 3-FPYPW was detected. Fig. 9 shows that the ML yield decreased slightly from 82.5 to 71.2% after four consecutive reaction cycles. Fig. 10 shows that the transmittance of FT-IR spectra and intensity of XRD patterns of the recycled 3-FPYPW catalyst were lower than that of the “fresh” counterpart, suggesting that the catalyst may have absorbed organic moieties during the reaction. In addition, the TGA patterns of the fresh and recovered 3-FPYPW catalysts were investigated. They confirmed the deposition of organic species onto the recycled catalyst (Fig. S2†), which possibly resulted in a loss of catalyst activity.

### 3.7 Reaction pathway for the conversion of fructose to ML

**Fig. 11:**
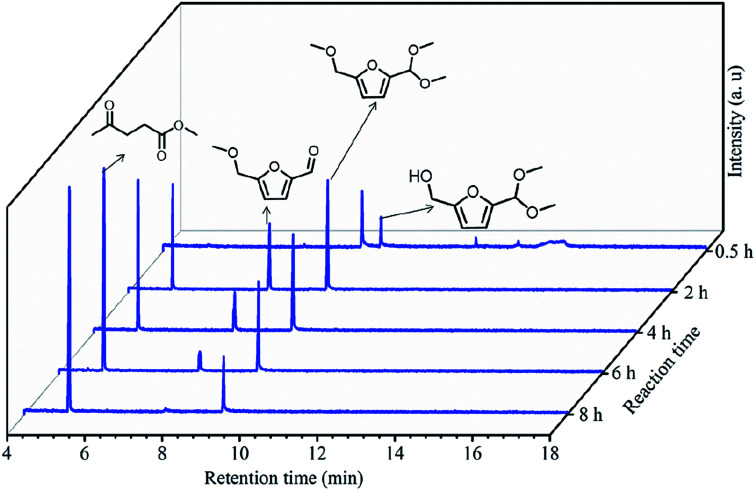
GC spectra at different reaction times for the conversion of fructose to ML.

**Scheme 2:**
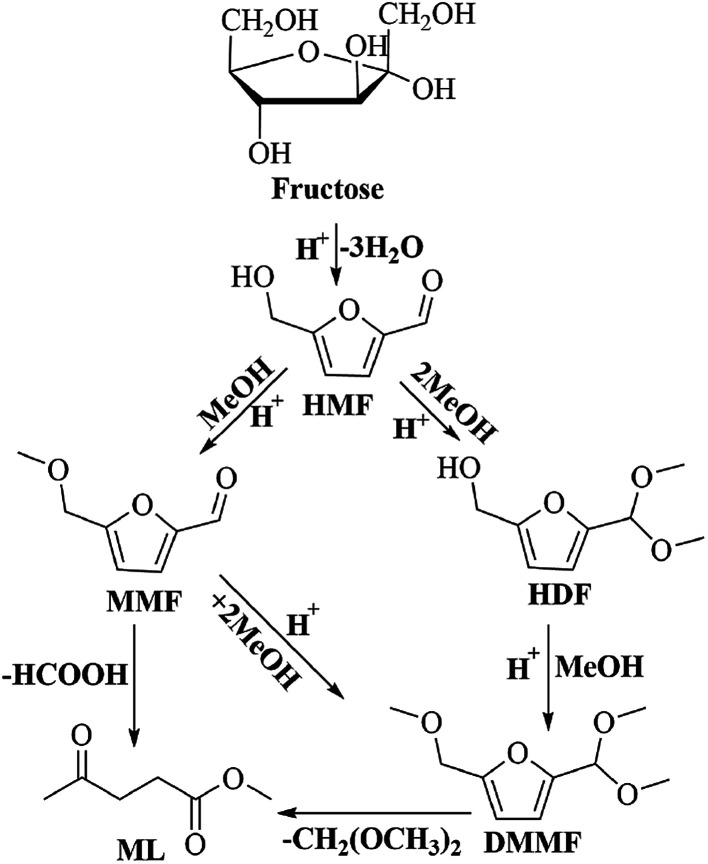
Proposed reaction pathway for the conversion of fructose to ML.

To understand the reaction pathway on the transformation of fructose to ML, experiments were undertaken at five reaction times (0.5 h, 2 h, 4 h, 6 h and 8 h) using fructose (0.2 mol L^−1^), 5 mL methanol, 3-FPYPW (0.1 mmol H^+^) and 120 °C, and GC-MS of the reaction mixtures undertaken (Fig. 11 and S3–S6). At 0.5 h, DMMF and 5-(dimethoxymethyl)-2-furanmethanol (HDF) were detected in the reaction mixture by GC-MS but MMF and ML were not found. ML, MMF and DMMF were detected when the reaction time was 2 h, but HDF disappeared. As the reaction time increased from 2 to 8 h, the amount of ML increased gradually, and MMF, DMMF decreased gradually. In addition, it has been reported that HMF is an intermediate in the degradation of fructose to ML.^31^ According to our experimental findings and the literature, the reaction pathway for the acid-catalyzed product of ML from fructose in methanol is proposed and the results are shown in Scheme 2. First, the loss of three water molecules from fructose *via* 3-FPYPW catalysis can yield HMF. Second, the hydroxyl or carbonyl group of HMF is attacked by a methanol molecule to form MMF or HDF, and reacts further with methanol to form DMMF. Lastly, the intermediate products MMF and DMMF are converted to ML over 3-FPYPW. In summary, fructose is first hydrated to HMF, followed by interaction with methanol to form ML, which is carried out efficiently in methanol under mild conditions using 3-FPYPW catalyst.

## 4. Conclusions

A series of HPW-based hybrids were prepared using a solvothermal method. These hybrids were applied for the conversion of fructose to ML in a single pot. The total acid density of the catalyst was measured by the sodium ion-exchange method. The total acid density of 3-FPYPW was higher than that of 3-MeFPYPW, 3-MeOPYPW or HPYPW. Among these catalysts, 3-FPYPW displayed superior catalytic activity for the synthesis of ML from fructose, and a 82.5% yield of ML was achieved using fructose (0.2 mol L^−1^), 5 mL methanol, and 0.1 mmol H^+^ for 10 h at 120 °C, which might be ascribed to the relatively higher acidity of 3-FPYPW. A reaction pathway for the conversion of fructose to ML was proposed. In addition, the 3-FPYPW catalyst could be reused for at least four cycles with only a slight decrease in its activity.

## Conflicts of interest

There are no conflicts to declare.

## Supplementary Material

RA-008-C8RA02278J-s001

## References

[cit1] Li H., Fang Z., Luo J., Yang S. (2017). Appl. Catal., B.

[cit2] Liu B., Zhang Z. H. (2016). ACS Catal..

[cit3] Liu B., Zhang Z. H. (2016). ChemSusChem.

[cit4] Mika L. T., Cséfalvay E., Németh Á. (2018). Chem. Rev..

[cit5] Hu L., Xu J. X., Xu S. Y., He A. Y., Tang X., Liu L., Xu J. M., Zhao Y. J. (2018). ACS Catal..

[cit6] Li D. F., Ni W. X., Hou Z. S. (2017). Chin. J. Catal..

[cit7] Li C. Z., Cai H. L., Zhang B., Li W. Z., Pei G. G., Dai T., Wang A. Q., Zhang T. (2015). Chin. J. Catal..

[cit8] Abbas S. N., Mok K. H., Rashid N., Xie Y., Ruether M., O'Brien J., Akhtar M. (2016). Bioorg. Chem..

[cit9] Li H., Yang S., Saravanamurugan S., Riisager A. (2017). ACS Catal..

[cit10] Kamran M. (2018). Renewable Sustainable Energy Rev..

[cit11] Zhang J., Wu S. B., Li B., Zhang H. (2012). ChemCatChem.

[cit12] Zhou L. P., Zou H. J., Nan J. X., Wu L., Yang X. M., Su Y. L., Lu T. L., Xu J. (2014). Catal. Commun..

[cit13] vom Stein T., Meuresch M., Limper D., Schmitz M., Hölscher M., Coetzee J., Cole-Hamilton D. J., Klankermayer J., Leitner W. (2014). J. Am. Chem. Soc..

[cit14] Díaz-Rodríguez A., Borzęcka W., Lavandera I., Gotor V. (2014). ACS Catal..

[cit15] Gupta D., Saha B. (2017). Catal. Commun..

[cit16] Tang X., Li Z., Zeng X. H., Jiang Y. T., Liu S. J., Lei T. Z., Sun Y., Lin L. (2015). ChemSusChem.

[cit17] Peng L. C., Lin L., Li H., L Yang Q. (2011). Appl. Energy.

[cit18] Saravanamurugan S., Riisager A. (2013). ChemCatChem.

[cit19] Zhao S. Q., Xu G. Z., Chang C., Fang S. Q., Liu Z., Du F. G. (2015). Catalysts.

[cit20] Njagi E. C., Genuino H. C., Kuo C. H., Dharmarathna S., Gudz A., L Suib S. (2015). Microporous Mesoporous Mater..

[cit21] Yu F., Zhong R. Y., Chong H., Smet M., Dehaen W., Sels B. F. (2017). Green Chem..

[cit22] Liu Y., Liu C. L., Wu H. Z., Dong W. S. (2013). Catal. Lett..

[cit23] Lai F. J., Luo J., Jiang D., Su T. C., Zhang F. (2018). J. Chem. Technol. Biotechnol..

[cit24] Liu B., Zhang Z. H., Deng K. J. (2012). Ind. Eng. Chem. Res..

[cit25] Chen J. Z., Zhao G. Y., Chen L. M. (2014). RSC Adv..

[cit26] Qu Y. S., Huang C. P., Song Y. L., Zhang J., Chen B. H. (2012). Bioresour. Technol..

[cit27] Wang Z. W., Li H., Fang C. J., Zhao W. F., Yang T. T., Yang S. (2017). Energy Technol..

[cit28] Liu T. T., Li Z. L., Li W., Shi C. J., Wang Y. (2013). Bioresour. Technol..

[cit29] Hernández-Cortez J. G., Martinez L., Soto L., López A., Navarrete J., Manríquez M., Lara V. H. (2010). Catal. Today.

[cit30] Thapa I., Mullen B., Saleem A., Leibig C., Baker R. T., Giorgi J. B. (2017). Appl. Catal., A.

[cit31] Lv G. Q., Deng L. L., Lu B. Q., Li J. L., Hou X. G., Yang Y. X. (2017). J. Cleaner Prod..

