# Supplementary material for: Phosphotungstic acid heterogenized by assembly with pyridines for efficient catalytic conversion of fructose to methyl levulinate
Source: RSC Adv. 2018 May 4;8(30):16585–92. doi: 10.1039/c8ra02278j (PMC9080306; doi:10.1039/c8ra02278j)
Supplement: RA-008-C8RA02278J-s001 [file RA-008-C8RA02278J-s001.pdf]

Electronic Supplementary Material (ESI) for RSC Advances.
This journal is © The Royal Society of Chemistry 2018

# Electronic supplementary information

## Phosphotungstic acid heterogenized by assembly with pyridine for efficient catalytic conversion of fructose to methyl levulinate

Chengjiang Fang[a], Yan Li[a], Wenfeng Zhao[a], Weibo Wu[a], Hu Li[a]*, Chao He[b], Song Yang[a]*

[a] *State Key Laboratory Breeding Base of Green Pesticide & Agricultura Bioengineering, Key Laboratory of Green Pesticide & Agricultural Bioengineering, Ministry of Education, State-Local Joint Laboratory for Comprehensive Utilization of Biomass, Center for Research & Development of Fine Chemicals, Guizhou University, Guiyang, Guizhou 550025, China*

[b] *School of Environmental Science and Engineering, Sun Yat-sen University, Guangzhou 510006, P. R. China*

* Corresponding authors:

E-mail: jhzx.msm@gmail.com (SY); hli13@gzu.edu.cn (HL)

Tel.: +86(851)8829-2171. Fax: +86(851)8829-2170

**Table of Contents:**

**Fig. S1** Effect of reaction time on the synthesis of ML from fructose

**Fig. S2** TGA patterns of fresh and recycled 3-FPYPW catalysts after four cycles

**Fig. S3** MS spectrum of methyl levulinate

**Fig. S4** MS spectrum of 2-(dimethoxymethyl)-5-(methoxymethyl)furan

**Fig. S5** MS spectrum of 5-methoxymethylfurfural

**Fig. S6** MS spectrum of 5-(dimethoxymethyl)-2-furanmethanol

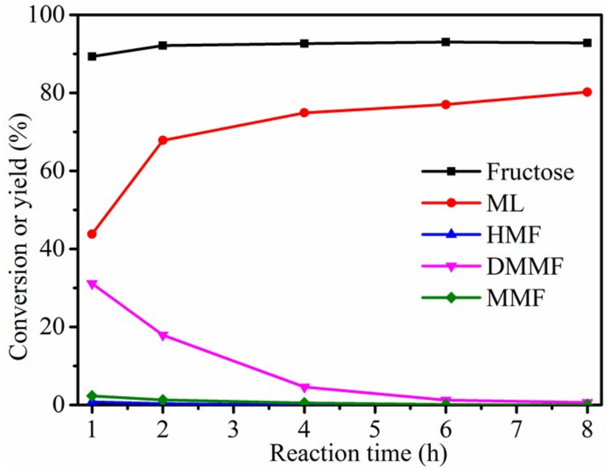


**Fig. S1** Effect of reaction time on the synthesis of ML from fructose. Reaction conditions: 0.2 mol/L fructose, 5 mL methanol, 3-FPYPW (0.1 mmol $H^+$), and 140 ºC.

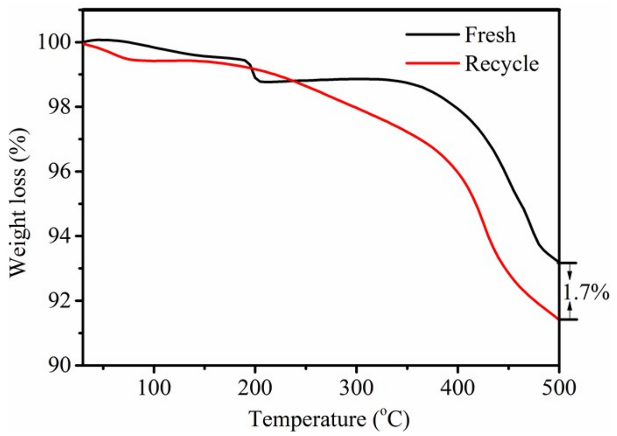


**Fig. S2** TGA patterns of fresh and recycled 3-FPYPW catalysts after four cycles

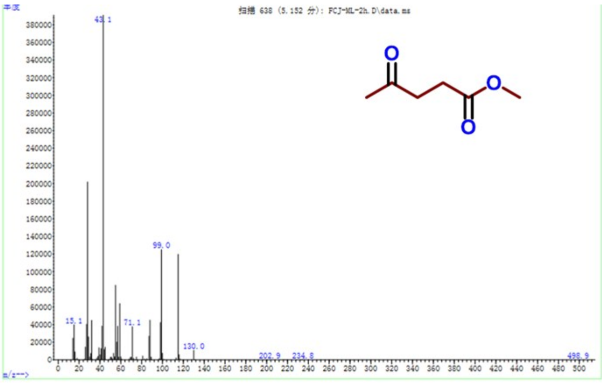


**Fig. S3** MS spectrum of methyl levulinate

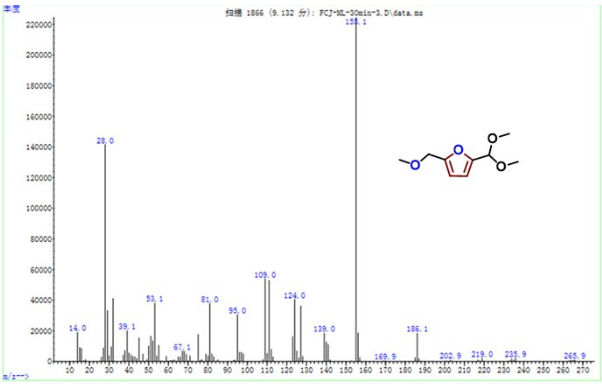


**Fig. S4** MS spectrum of 2-(dimethoxymethyl)-5-(methoxymethyl)furan

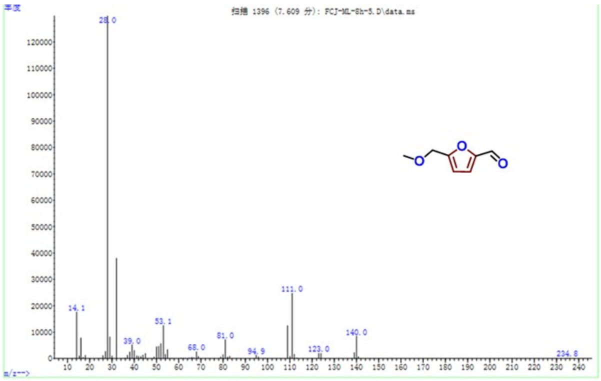


**Fig. S5** MS spectrum of 5-methoxymethylfurfural

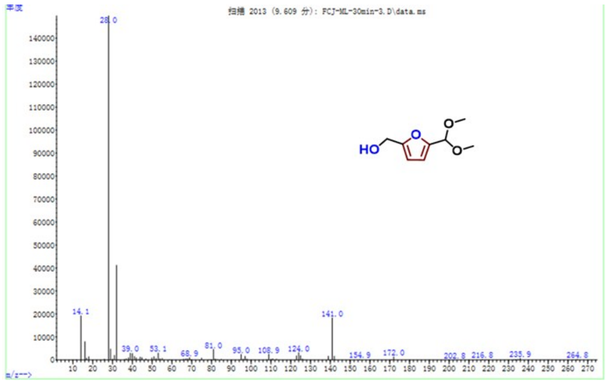

**Fig. S6** MS spectrum of 5-(dimethoxymethyl)-2-furanmethanol
